# Comparison of Efficacy of Autologous Platelet Rich Plasma Therapy With 5% Topical Minoxidil Spray in Treating Alopecia Areata: A Head-to-Head Assessment of Novel Approaches

**DOI:** 10.7759/cureus.61878

**Published:** 2024-06-07

**Authors:** Anjum Muhammad, Sadaf Saleem, Shumaila Khan, Gurnam Virdi, Samina Arshad, Sohail Muhammad, Muhammad T Younas, Afshan Saeed, Deeba S Khan, Ateka Ikram

**Affiliations:** 1 Dermatology, Combined Military Hospital, Kohat, PAK; 2 Dermatology, Sheikh Zayed Hospital, Lahore, PAK; 3 Dermatology, Pakistan Academy of Aesthetic Dermatology (PAAD), Islamabad, PAK; 4 Dermatology, Academy of Aesthetic Medicine United Kingdom, Birmingham, GBR; 5 Dermatology, Combined Military Hospital, Lahore, PAK; 6 Dermatology, Mian Rashid Hussain Shaheed Hospital, Nowshehra, PAK; 7 Medicine, Russells Hall Hospital, Dudley, GBR; 8 Dermatology, Pak-Emirates Military Hospital, Rawalpindi, PAK; 9 Dermatology, Jinnah Memorial Hospital, Rawalpindi, PAK; 10 Internal Medicine, Pakistan Ordnance Factory (POF) Hospital, Islamabad, PAK

**Keywords:** prp vs minoxidil, loss of scalp hair, 5% topical minoxidil, platelet-rich plasma/ prp, alopecia areata (a.a.) treatment

## Abstract

Background: Alopecia areata (AA) remains one of the most challenging afflictions encountered in dermatology clinics. It is characterized by an autoimmune-mediated inflammatory process of and around hair follicles, causing reversible, non-scarring hair loss. With the ongoing search for optimal treatment strategies, the potentially positive role of autologous platelet-rich plasma (PRP) therapy as well as minoxidil has been reported in various studies; however, the comparison of the two treatment modalities is largely underexplored. This research aims to compare and assess the effectiveness of intralesional PRP with topical minoxidil therapy in AA to identify efficacious management options amongst the newly described treatment modalities.

Methodology: The research work was conducted over four months and included 40 (31 males and 9 females) patients suffering from alopecia areata. They were divided into Group A, which was administered monthly autologous PRP injections, while Group B was given daily topical 5% minoxidil therapy. In group A, four treatments of PRP were given, each one month apart. While in group B, daily topical minoxidil spray was administered for the same duration. The alopecia areata severity grade was recorded by employing the "Severity of Alopecia Tool" (SALT) scoring system. The pre- and post-treatment SALT scores were noted and compared at each monthly visit.

Results: The study comprised nine (22.5%) female and 31 (77.5%) male patients. At the beginning of the study and after one month of treatment, the difference in the SALT score was not statistically significant between the two groups, suggesting that both interventions had similar effects during the early stages of the treatment. At two months, a statistically significant difference emerged (p-value 0.037), indicating that a more significant fall in the SALT score was observed with autologous PRP treatment compared to topical minoxidil. After four months, a highly significant difference was noted between the two groups (p-value <0.0001), implying that intralesional PRP therapy led to a far more significant decrease in the SALT score compared to topical minoxidil therapy.

Conclusion: Monthly intralesional autologous PRP therapy for four months manifests better outcomes in alopecia areata than daily 5% topical minoxidil therapy.

## Introduction

Alopecia areata (AA) remains a well-recognized autoimmune dermatological ailment. The abrupt onset of hair loss in patches is its symptomatic hallmark, mostly involving the localized areas of the scalp, face, or any other part of the skin. With an estimated lifetime prevalence of 2.1%, affecting millions of patients worldwide, AA affects both children and adults, with an equal male and female preponderance [1]. AA is thought to emanate due to a complex set of interactions amongst genetic, environmental, and immunological elements; however, the exact etiopathological basis remains debated [2].

AA exerts a considerable amount of psychosocial morbidity on the affected patients [1], and the current treatment strategies are aimed at suppressing the autoimmune response to stimulate and maintain hair growth [2]. Although the use of topical and intralesional steroids is one of the most productive treatment options to combat localized AA, they come with a host of side effects, of which local skin atrophy, telangiectasia, and hypopigmentation are the most common [3].

With the ongoing search for optimal treatment strategies for AA, there has been enthusiasm for using autologous platelet-rich plasma therapy (PRP) in hair restoration and maintenance. Numerous studies have employed and reported the beneficial role of PRP in hair rejuvenation [4,5]. There is growing evidence in favor of using PRP for androgenetic alopecia treatment and hair maintenance [4]. Similarly, topical minoxidil has extensively been studied and utilized in different types of alopecia. It acts to reduce hair loss and increase hair growth by shortening the telogen phase, increasing DNA synthesis inside anagen bulbs, stimulating the secondary hair germ cells in the telogen follicles, and causing a robust shift to the anagen phase, according to studies [6]. It comes only naturally that clinical dermatologists perform a head-to-head comparison of different treatment modalities to identify the optimal treatment option for the treatment of AA. Due to the multiple side effects ascribed to intralesional (and topical) steroids, PRP and topical minoxidil, both, might seem like attractive options for patients and clinicians alike. That being said, both treatment modalities come with their own set of merits and demerits. While a few studies have conducted a comparison of the efficiency of topical minoxidil and intralesional PRP in the management of AA [7], the matter is still relatively underreported. The purpose of this therapeutic trial was to assess and compare intralesional PRP with topical minoxidil in the management of AA to fill and improve this knowledge gap.

## Materials and methods

This quasi-experimental research was carried out at the outpatient department of dermatology in a tertiary healthcare facility over four months (November 2023 to February 2024) after due approval by the institutional ethical review (A/8/EC/55/23 Oct 2023).

Inclusion criteria: Patients presenting with AA and willing for autologous intralesional PRP (IL PRP) treatment and 5% topical minoxidil treatment and able to maintain a regular follow-up were inducted into the study.

Exclusion criteria: The individuals who presented with hair loss other than AA, having hematological, endocrinological (thyroid, DM, PCOS, Cushing disease/syndrome, or any signs and symptoms that were suggestive of any acute or chronic hormonal abnormalities), those with any coagulation, nutritional and chronic dermatological disorders that contributed to hair loss, patients who received any hair medications/interventions in the past six months, those presenting with any active site infection, known allergy to topical minoxidil or other factors that made them unfit for IL PRP or minoxidil and those who were not able to get a platelet count of ≥0.1 million/mL (a working definition of PRP by Marx et al.) were also excluded from the study [8].

Following informed, explicit consent, 40 individuals were inducted into the research. Randomization was carried out by sequentially numbered opaque envelopes generated from a random numbers table into two groups of 20 volunteers each. Each volunteer was given a number at enrolment, which defined a study treatment modality assignment (IL-PRP or 5% minoxidil spray). Using this sampling strategy, patients were selected and stratified into two groups, i.e., group A (intralesional autologous PRP, injected intra-dermally and subcutaneously, 0.1 ml injection 1 cm apart via insulin syringe [4]) and group B (daily topical 5% minoxidil spray with the brand name Minoxin Plus by Brooks Pharma Pakistan, and massaged for 2-3 minutes on the affected area at night [6]).

Autologous PRP was prepared under strict aseptic measures with an anticoagulant (Citrate-phosphate-dextrose solution with adenine; CPDA) mixed with fresh 15 mL of venous blood from medial cubital venipuncture. As per the directions of the Technical Manual of the American Association of Blood Banks [9], centrifugation of venous blood was carried out for 15 minutes (soft spin centrifugation at 2000 rotations per minute) first and then for another 10 minutes of hard spin centrifugation (4000 rotations per minute). This procedure aimed to get a platelet count of ≥0.1 million/mL in a total of 5 mL of PRP, a working definition of PRP by Marx et al. [8].

Using the SALT scoring system, the AA grade recorded pre-treatment at every visit and the culmination of the research. To calculate the SALT score, the scalp area is ramified into four equal parts: the vertex, which is assigned 40% (0.4) of the scalp area, while the right and left sides of the scalp are assigned 18% (0.18) of the scalp surface area on each side. The posterior aspect of the scalp is given 24% (0.24) of the scalp surface area [9]. The percentage of hair loss in any of the four areas is multiplied by the percentage of the respective surface area of the scalp. The final score is calculated by adding all hair loss percentages in the respective scalp areas mentioned above [9].

Different variables of the participants were recorded for data collection purposes. The data analysis was done by employing IBM Corp. Released 2015. IBM SPSS Statistics for Windows, Version 23.0. Armonk, NY: IBM Corp. Baseline variables were analyzed descriptively using frequencies and percentages for qualitative variables, and the mean with SD was calculated for quantitative variables. The independent t-test and Mann-Whitney U test were applied for the comparison of quantitative variables and to compare the non-normally distributed variables, respectively. A p-value of less than 0.05 was regarded as significant.

## Results

Our research involved a total of 40 individuals. Thirty-one (77.5%) were male (mean age of 31.31±6.81 years) and nine (22.5%) were female (with a mean age of 37.88±13.61 years). All of the patients were of eastern Mediterranean Pakistani ethnicity.

More than half of the patients reported an AA duration of more than six months. The majority of the patients denied any family history of AA, the presence of autoimmune disease, or any history of atopy. These details are shown in Table 1.

**Table 1:** The baseline demographic characteristics of the patients

Parameters		n (%)
Gender	Male	31 (77.5%)
	Female	9 (22.5%)
Duration of the Disease	<6 Months	18 (45.0%)
	>6 Months	22 (55.5%)
History of Atopy	Yes	8 (15.5%)
	No	32 (82.5%)
Family History of Alopecia Areata	Yes	2 (5.0%)
	No	38(95.0%)
History of Autoimmune Disease	Yes	4 (10.0%)
	No	36 (90.0%)

The comparison between the two management modalities, Group A (PRP) and Group B (Topical minoxidil) in the management of AA is shown in Table 2.

**Table 2:** Comparison of platelet-rich plasma and topical minoxidil in the treatment of alopecia areata in terms of SALT score

SALT Score	Groups		p-Value
	Group A, n=20 (Platelet Rich Plasma) (Mean±SD)	Group B, n=20 (Minoxidil) (Mean±SD)	
Baseline	6.41± 1.01	6.34 ± 0.83	0.799
At 1 Month	5.62 ± 0.78	5.82 ± 0.92	0.451
At 2 Months	4.47 ± 0.81	4.99 ± 0.74	0.037
At 3 Months	3.33 ± 0.62	3.91 ± 0.51	0.003
At 4 Months	2.07 ± 0.57	2.80 ± 0.41	<0.0001

At the beginning of the research, no statistically significant difference was seen between the two modalities in terms of the decrease in the SALT score values, implying that both groups started with a comparable severity of AA. After one month of treatment, the two groups still did not show any significant difference, indicating that both modalities had somewhat similar effects during the early stages of treatment. At two months, a statistically significant difference emerged, indicating that PRP displayed a more significant fall in the SALT score in comparison to minoxidil. At the culmination of the research period, the difference shown by the two modalities became far more significant, suggesting that IL PRP led to a higher reduction in the SALT score compared to topical minoxidil.

SALT Score differences at four months compared to baseline values between PRP and topical minoxidil in AA are shown in Table 3.

**Table 3:** SALT score difference at the end of the study in comparison to baseline between platelet-rich plasma and minoxidil in alopecia areata treatment.

Difference in the SALT score at the culmination of the treatment, compared to baseline	Groups		p-value
	Group A	Group B	
	4.34 ± 0.98	3.59 ± 0.86	0.007

The p-value of 0.007 implied a significant difference between PRP and minoxidil in terms of their SALT scores at the end of the study period, compared to baseline, favoring IL PRP as a better treatment option than topical minoxidil.

## Discussion

Alopecia areata is an autoimmune and inflammatory hair disorder that primarily affects the hair follicles but may also involve the nails in certain individuals [1]. Our study not only reports the potentially beneficial role of PRP and minoxidil in AA but also compares their relative efficacy over a prolonged period.

While PRP therapy may appear to have its own set of merits, namely a more favorable safety profile in terms of far lesser chances of cutaneous atrophy compared to steroids, it does come with its own set of demerits, such as pain, bruises, swelling, and visits to clinics to get the procedure done. Minoxidil, in a somewhat similar way, is easy to administer at home but does come with side effects like headaches, dermatitis, and hypertrichosis in non-treated areas, among other side effects. Some patients might find a compliance issue with its daily application [6]. Our study holds significance as the comparison of the two treatment modalities gives both clinicians and patients an insight into choosing a particular treatment modality to tailor AA management on an individual basis.

The rationale for using PRP for various types of alopecia areata lies in the regenerative qualities of platelets, which bring about the release of a host of cytokines and growth factors, which, in turn, contribute to hair follicle stimulation and tissue repair [5]. The very same principle has been employed in wound healing and androgenetic alopecia [4]. Minoxidil therapy, on the other hand, exerts its therapeutic effects by vascular vasodilation, shortening the telogen phase, increasing the DNA synthesis in anagen bulbs, and increasing the anagen phase [6]. With well-known side effects like skin irritation, headache, and hypertrichosis in non-treated areas ascribed to minoxidil use, the latter side effect is especially worrisome for female patients, causing significant compliance issues [6].

Of the 40 volunteers inducted in our study, the majority were young male patients in their late twenties and early thirties. More than half of our patients reported the duration of AA to be more than six months, while the severity of the ailment was mild, with the majority of the volunteers presenting with a single patch of AA. 

As mentioned earlier, the topical minoxidil and IL-PRP groups showed no statistically significant difference in the reduction in the SALT score in the earlier phases of our research. After one month of treatment, differences in SLAT scores between the two groups were non-significant, suggesting that both modalities had somewhat similar effects in the early stages of treatment. According to our observations, the positive results of topical minoxidil in AA treatment may be short-reaching. This is evident from the observation that, at two months, the PRP was found to have a more significant fall in the SALT score in comparison to topical minoxidil. This difference became more pronounced after a four-month interval, indicating that PRP led to a more substantial reduction in the SALT score compared to topical minoxidil. This can be explained by hypothesizing that the growth factors and cytokines delivered during PRP treatment exert a long-term beneficial effect, while minoxidil may be devoid of the same effects in AA. These observations are consistent with research by El Taieb et al., who reported better treatment outcomes with the PRP group [7]. Our finding regarding the effectiveness of autologous PRP in AA is also consistent with the research works of Balakrishnan et al. [3] as well as Altaf H et al. [10], who reported a higher reduction in SALT scores in patients who received PRP treatment. Although both of these studies compared IL-PRP with intralesional steroids in AA, they nevertheless testify to the efficacy of PRP in AA.

A systemic review in 2022 observed that a total of five studies reported the effectiveness of PRP as monotherapy in AA. The review, however, also stated that while PRP may be a promising treatment for AA in those having side effects with steroids, the utilization of PRP is yet to be standardized, and it may not be chosen as the first-line management strategy for autoimmune hair loss such as AA, as there is still a relative lack of high-quality evidence [11]. Our findings are contradicted by an early study where PRP was found to have no efficacy in AA [12]. The study, however, had a small sample size and provided only three sessions of PRP. A larger sample size gives more weight to our study. A small pilot study by A. Trink et al. also reported the efficacy of autologous PRP in alopecia areata; however, the comparison was done with intralesional steroids [13].

With the search for a better understanding of the etio-pathogensis as well as optimal management of AA still ongoing [14], our study attempts to report more efficacious options of the two relatively novel treatment modalities like PRP and minoxidil. According to our observations, while PRP may be more efficacious than topical minoxidil in AA, the combination of the two may be more beneficial to the patients as both treatments target different mechanisms of action. Further research is needed to ascertain this hypothesis.

The limitations of our study include not recording the incidence of side effects in both PRP and minoxidil arms. Recording and comparing the side effects and patient comfort level with both modalities would have given a better idea to both clinicians and patients. The reason we kept our focus on comparing the efficacy of PRP with minoxidil instead of looking into the side effect profiles of these two modalities was that both PRP and minoxidil are generally devoid of any significant side effects anyway [7,15], especially when they are used in limited-scale AA. Other limitations included the unblinded nature, a relatively smaller sample size, and a shorter duration of follow-up, as well as the possibility of the presence of inter-observer variability during the calculation of the SALT score.

## Conclusions

Intralesional autologous platelet-rich plasma therapy may represent an encouraging therapeutic option for individuals with alopecia areata, both as an adjunct or even first line, especially if the AA patients are suffering from untoward side effects of intralesional steroids, such as skin atrophy. Similarly, PRP is more efficacious than topical minoxidil therapy alone, although the combination of the two may potentially offer far better outcomes, especially when the individual presents with a low-grade AA. Customized treatment plans tailored according to availability, patient preferences and needs, the side effects profile from previous treatments, and the disease characteristics are important for an optimal therapeutic outcome in alopecia areata.
